# Differential activation of the lateral premotor cortex during action observation

**DOI:** 10.1186/1471-2202-11-89

**Published:** 2010-07-31

**Authors:** Sebastian Pilgramm, Britta Lorey, Rudolf Stark, Jörn Munzert, Dieter Vaitl, Karen Zentgraf

**Affiliations:** 1 Institute of Sport Science, Justus Liebig University Giessen, Kugelberg 62, 35394 Giessen, Germany; 2 Bender Institute of Neuroimaging, Justus Liebig University Giessen, Otto-Behaghel-Str. 10 H, 35394 Giessen, Germany; 3 Institute of Sport Science, University of Berne, Bremgartenstrasse 145, 3012 Bern, Switzerland

## Abstract

**Background:**

Action observation leads to neural activation of the human premotor cortex. This study examined how the level of motor expertise (expert vs. novice) in ballroom dancing and the visual viewpoint (internal vs. external viewpoint) influence this activation within different parts of this area of the brain.

**Results:**

Sixteen dance experts and 16 novices observed ballroom dance videos from internal or external viewpoints while lying in a functional magnetic resonance imaging scanner. A conjunction analysis of all observation conditions showed that action observation activated distinct networks of premotor, parietal, and cerebellar structures. Experts revealed increased activation in the ventral premotor cortex compared to novices. An internal viewpoint led to higher activation of the dorsal premotor cortex.

**Conclusions:**

The present results suggest that the ventral and dorsal premotor cortex adopt differential roles during action observation depending on the level of motor expertise and the viewpoint.

## Background

Humans are social individuals who interact with their environment. Thus, performance in certain situations benefits from understanding the actions of others. Walking through a crowded shopping mall, playing soccer in a team, or ballroom dancing are prime examples that illustrate the relevance of visual input for acting appropriately in social settings. Accordingly, our brain dedicates significant neural resources to the comprehension of observed actions.

The discovery of neurons that fire in the ventral and dorsal premotor cortex (BA 6) during both action execution and action observation [1,2] led to a functional reappraisal of what was formerly considered to be a motor area. The role of these so-called mirror neurons has become a topic of much debate among neuroscientists, psychologists, and philosophers, and there are wide-ranging interpretations of activity in the mirror-neuron system including the facilitation of viewing goal-directed actions [3], imitative behavior [4], action understanding [5], language development [6], and implementation of perception-action circuits [7].

Recent attention has focused on factors that may modulate neural activity in areas such as the premotor cortex during action observation [[8-12]; for a review see [13]]. For example, presented actions made with different effectors determined a somatotopically activation of the premotor cortex similar to that of the classical motor cortex homunculus [14,15]. Regarding expertise, Cross et al. [8]have revealed that active training of dance sequences compared to sequences without training, leads to a greater activation of the ventral premotor cortex. Calvo-Merino et al. [9] demonstrated an effect of motor expertise on neural activation within the ventral premotor area (remapped according to Rizzolatti et al. [5]) for expert dancers. They have also shown stronger activation in the inferior parietal and cerebellar regions when observing dance videos, suggesting that the action observation network is more extended than previous classical primate studies have suggested [16-18]].

There is obviously no doubt that participating in a dancing course is associated with both first-person and third-person visual experience. However, active training is first and foremost associated with gaining first-person visual experience in a specific setting. When it comes to dance experts and novices, the role of the viewpoint in action observation is still unclear. In all the available dance studies using functional magnetic resonance imaging, videos were presented from an external viewpoint using cameras in a fixed position during capturing. It would be more natural to let participants observe videos recorded from an internal viewpoint with a moving camera. The internal viewpoint depicts a participant's view of a ballroom dance setting. Jackson and colleagues [19] conducted an experiment comparing neural correlates during the observation of videos of simple hand and foot movements from an internal and an external visual viewpoint. Their results demonstrated increased activation in the dorsal premotor cortex during action observation in the internal condition compared to the external. This suggests that dorsal premotor cortex activation during action observation might be related not only to motor expertise or motor familiarity but also to the internal visual viewpoint.

As stated above, several studies have investigated cerebral activity within the human action observation network [8-12,19] consisting of the premotor cortex and inferior parietal lobe [20]. In particular, their results suggest a differential impact of either motor expertise or viewpoint on the ventral and the dorsal premotor cortex. However, none of these studies have tested this possible functional dissociation within one single design. Therefore, we applied a 2 × 2 factorial within-subject design with two levels of dance expertise (novices and experts) and two types of ballroom dance videos (from an internal and an external viewpoint). Drawing on former results, we hypothesized a differential influence of the two experimental variables motor expertise and viewpoint on premotor activation sites, with motor expertise being associated with increased activation of the ventral premotor cortex and an internal viewpoint being associated with increased activation of the dorsal part of the premotor cortex.

## Results

### Recognition Test

After the scanning session, participants completed a recognition test. Experts had an average of 80.2% (*SD *= 13.22) correct responses on the recognition test compared to73.2% (*SD = *9.52) in the novices. One-sample *t *tests showed that the performance of both groups differed significantly from chance (*t(15)*_*expert*s _= 9.14, *p_experts _*< 0.001; *t(15)_novices _*= 9.77, *p_novices _*< 0.001).

A 2 (expertise: experts vs. novices) × 2 (viewpoint: internal vs. external) repeated-measures ANOVA revealed a significant main effect of viewpoint (*F(1, 30) *= 25.96; *p *< 0.001), but no significant main effect of expertise (*F(1, 30) *= 3.82; *p *= 0.06). There was no significant interaction (*F(1, 30) *= 0.474; *p *= 0.496).

### Functional Magnetic Resonance Imaging Data

#### Observation of Ballroom Dancing Scenes

To detect which brain regions were activated in the mere observation of dance movements, we first contrasted each observation condition with the corresponding scrambled video. The resulting *t *contrasts were fed into a conjunction analysis. This analysis of all observation conditions revealed significant activation in regions previously shown to be involved in action observation. Brain areas activated by observing dancing sequences were the inferior parietal lobe of the right hemisphere, the middle occipital cortex of the left hemisphere, the left superior parietal cortex, the fusiform gyrus of the left hemisphere, the dorsal premotor cortex of both hemispheres, as well as the left cerebellum (α < .05, FWE-corrected). These results are consistent with a number of studies demonstrating the involvement of these areas in action observation [8,9]). All results are summarized in Table 1.

**Table 1:** Brain regions activated by action observation (conjunction of all observation conditions compared to scrambled videos)

	l/r	Coordinates of max *t *value			*t*
					
		*x*	*y*	*z*	value
Inferior parietal lobe	r	48	-57	9	11.24

Middle occipital cortex	l	-48	-81	0	9.59

Superior parietal cortex	l	-33	-45	57	7.63

Fusiform gyrus	l	-42	-51	-15	6.84

Dorsal premotor cortex	r	45	0	51	6.53

Occipital gyrus	r	24	-78	36	6.30

Dorsal premotor cortex	l	-30	-9	54	5.89

Cerebellum	l	-12	-78	-42	5.52

Dorsal premotor cortex	l	-9	-3	66	5.10

MNI coordinates, *p *< 0.05 (FWE-corrected).

### Effects Related to Motor Expertise

In a first step, we identified which premotor region was associated more strongly with motor expertise. For this purpose, we calculated a two-sample t-test of the contrast ([*internal viewpoint (IV) - scrambled internal viewpoint (SIV)] + [external viewpoint (EV) - scrambled external viewpoint (SEV)]*) comparing experts minus novices.

Region-of-interest (ROI) analyses revealed a significant activation of the left ventral premotor cortex. No activation was found within the dorsal part of the premotor area. The masks for our ROI analyses resulted from a splitting of a probabilistic mask of the premotor cortex (BA6) at *Z *= 50 into a ventral and a dorsal premotor cortex, as suggested by Rizzolatti et al. [5]. All results are summarized in Table 2. The opposite contrast, which would show higher activation within novices compared to experts, revealed no significant activation differences within the predefined ROIs.

**Table 2:** Brain regions activated by main-effect expertise and viewpoint

	l/r	Coordinates of max *t *value			*t*
					
Experts > Novices		*x*	*y*	*z*	value
Ventral premotor cortex	l	-57	6	33	3.41

					

**Internal > External**					

Dorsal premotor cortex	l	-24	-6	51	4.11

Dorsal premotor cortex	l	-18	-6	63	3.63

Dorsal premotor cortex	r	24	-3	60	3.84

Ventral premotor cortex	l	-12	-18	45	4.04

MNI coordinates, *p *< 0.05 (FWE-corrected).

### Effects Related to Viewpoint

To identify the part of the premotor area associated with higher activation when observing a dancing scene from an internal viewpoint, we computed the contrast *(IV-SIV) - (EV-SEV)*. Activation differences resulting from an internal viewpoint revealed significant activation within the dorsal premotor cortex (bilateral) as well as within the left superior ventral premotor cortex.

Again, the opposite contrast *(EV-SEV) - (IV-SIV) *revealed no significant activation differences in the ROIs (see Table 2).

### Interaction of Expertise and Viewpoint

In order to detect which areas were activated in experts when observing dancing scenes with an internal viewpoint, we calculated the contrast *([IV-SIV] - [EV-SEV]_experts _- [IV-SIV] - [EV-SEV]_novices_)*. No significant differences in activation sites could be found.

## Discussion

**Figure 1:**
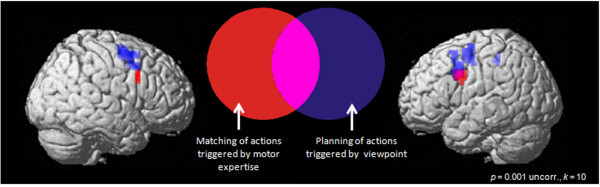
**Differential Activation of the Premotor Cortex (BA 6/44)**. Differential activation dependent on the factors expertise (red) and viewpoint (blue) rendered on the same average brain images. Voxels activated in both conditions are displayed in purple.

Using functional magnetic resonance imaging, we investigated the role of viewpoint and motor expertise on neural activation of the lateral premotor cortex during the observation of dance movements. Participants with different levels of expertise (dancing novices and experts) observed ballroom dance sequences from an internal and an external viewpoint. First, we replicated the importance of the action observation network in the observation of dance movements [8-12]. Second, we found that motor experts reveal increased activation in the ventral part of the premotor cortex. Third, we demonstrated that activation in the left ventral as well as in the dorsal premotor cortex (bilateral) differs according to whether internal-viewpoint or external-viewpoint videos are being observed (see Figure 1). These findings not only deliver further evidence supporting the importance of the dorsal premotor cortex in action observation but also indicate a functional dissociation of the two areas.

A broad body of evidence supports the notion that the human brain contains specialized parietal-premotor circuits that are activated when observing and understanding the actions of others [8-12,19,21]. Furthermore, Calvo-Merino et al. [9] have confirmed that the cerebellum is also part of a broader action observation network. Our results showed increased activation within a network of brain areas consisting of premotor, parietal, and cerebellar regions when participants observe ballroom dance scenes irrespective of motor expertise and viewpoint. One major idea underling both the above-mentioned studies and the present results is that action observation and the resultant understanding of actions is not just a visual process, but also a process related to motor representations that is associated with activation in areas responsible for the observer's motor repertoire. According to this notion, action understanding results particularly from a mechanism that maps an observed action onto a motor representation of that action [17,22-25].

The premotor cortex does not just play a pivotal role in motor preparation, motor execution, action planning, and decision-making for action selection. It is pivotal in action observation as well [26-29]. The premotor cortex of primates is also thought to play an important role in the sensory guidance of motor behavior [30,31]. Anatomically, it is divided into two major parts, the ventral and the dorsal premotor area. Alongside cytoarchitectonic differences [32], the two subareas also differ in their anatomical interconnections. For example, the dorsal premotor cortex receives its main input from the dorsal part of the dorsolateral prefrontal cortex and the superior parietal cortex [32,33], whereas the ventral premotor cortex receives its main input from the ventral part of the dorsolateral prefrontal cortex as well as from the inferior parietal lobule [32,34]. These anatomical considerations suggest that the dorsal and the ventral premotor cortex may even be distinct on a functional level.

Within this framework, the present data do reveal a functional differentiation between the ventral and the dorsal premotor cortex. Increased activation in the ventral portion of the premotor cortex in participants with greater motor expertise is in line with the results of a study conducted by Calvo-Merino et al. [9]. This demonstrated that expert dancers reveal increased activation within the ventral part of the premotor cortex. Another study [8] induced expertise through a short period of physical training in the observed dance sequence. Participants in this study also showed increased activation in the ventral premotor cortex while observing the trained sequences. Thus, activation in the ventral part of the premotor cortex seems to reflect motor expertise. Hoshi and Tanji [29] have reasoned that an important function of the ventral premotor cortex is to match motor acts with sensory inputs. Matching an observed act with an actually executed movement seems to be accomplished by the mirror neuron system [35]. This mirror process can be categorized as a variant of direct matching between the sensory information and a motor act. In this context, an expert can be viewed as a person who has gained multiple physical experiences in her or his domain of expertise. This experience enhances the development of specific internal sensorimotor representations [36]. Hence, it is due to their experience that experts display matching between the specific action-oriented sensory information and the associated motor act. For example, an expert dancer knows what kind of action has to be performed when observing the spatial limb positions of a dancing partner. Within the present study, the increased activation of the experts' ventral premotor cortex might therefore reflect their ability to match visuospatial information onto their own motor representations [29]. In addition, contrasting the internal with the external viewpoint also revealed activation within the left ventral premotor cortex. In case, ventral premotor activity reflects the direct matching of visuospatial information on motor representations, this result supports the fact that such a matching occurred rather when observing scenes from an internal viewpoint, i.e. when the observed scene is to a higher degree related to a person's own action, than when observing scenes from an external viewpoint.

Interestingly, the present data revealed increased activation in the dorsal part of the premotor cortex, particularly for dancing scenes observed from an internal viewpoint. More precisely, this activation cluster captured a large part of the dorsal premotor cortex and the adjoining superior half of the ventral premotor cortex. These findings are in line with the results of Jackson et al. [19], who have demonstrated increased activation within the dorsal premotor cortex while participants observe intransitive actions from an internal viewpoint. In a landmark study, Boussaoud and Wise [37] have demonstrated that neurons within the dorsal part of the premotor cortex are associated more strongly with representing the motor significance of visual stimuli. In the present study, the greater activation of the dorsal part of the premotor cortex in the internal-viewpoint condition supports the idea that different viewpoints reflect different levels of motor significance. Sensory information gained from an internal viewpoint encourages the planning of an action. Hoshi and Tanji [29] have argued that ventral premotor cortex activation is relevant for matching actions directly with sensory input, whereas dorsal premotor cortex activation is more relevant for planning actions. Thus, we would suggest that the internal viewpoint might trigger a form of movement planning that is reflected by dorsal premotor cortex activation due to the natural and familiar situation. Second, the increased activation of the ventral premotor cortex among experts indicates that familiarity with observed scenes and the associated visuospatial features may trigger a process by which the observed motor acts and their motor coordinates are matched with own motor representations [29].

A possible flaw in our interpretation would be if the activation differences we found within the premotor cortex resulted from the fact that in the internal viewpoint feet and legs were presented to a greater extent, than in the external viewpoint. This might trigger a somatotopical activation of the dorsal PMC by the internal viewpoint, whereas the external viewpoint would activate the whole PMC. For example, Buccino et al. [14] elucidated that the observation of movements of body parts activates the PMC in a somatotopically organized manner. Nevertheless, several arguments would allow us to rule out that the present results were determined by the body parts which were seen in the dancing scene. Foremost, in both experimental conditions hands, arms, legs, feet as well as the upper part of the body are seen. Therefore, the conditions do not differ with respect to body parts. Secondly, given that the external viewpoint involves observation of the whole body, it could be argued that activation in a somatotopical manner should have revealed an increased activation of the whole PMC. In the present study contrasting the external and the internal view did not reveal any significant activation site within the PMC.

## Conclusions

In conclusion, we have demonstrated that motor expertise is associated with stronger ventral premotor cortex activation when viewing familiar dance movements. Viewpoint, however, seems to affect dorsal premotor cortex activation more strongly. Therefore, our data provide evidence that dorsal and ventral premotor areas are differentially activated during action observation depending on different levels of expertise and on different visual viewpoints.

## Methods

### Participants

Thirty-six (18 experts) right-handed [35] women (M_experts_= 28.94 years of age, SD_experts_= 10.9; M_novices _= 24.13 years of age, SD_novices _= 7.1) participated in the study. Four participants (two experts) had to be excluded due to technical problems. All participants were healthy and nonmedicated. We restricted the sample to women, because the videos in the internal condition were recorded from a female's viewpoint. Novices had little experience in ballroom dancing: They had attended no more than four dance classes in their life, and the last class was at least 4 years ago. Experts were all skilled dancers with at least 5 years of continuous dance experience. Dancing expertise was assessed by a questionnaire.

The study was approved by the ethical committee of the German Psychological Association (*Deutsche Gesellschaft für Psychologie*), and all participants gave their informed written consent in accordance with the Declaration of Helsinki.

### Procedure

**Figure 2:**
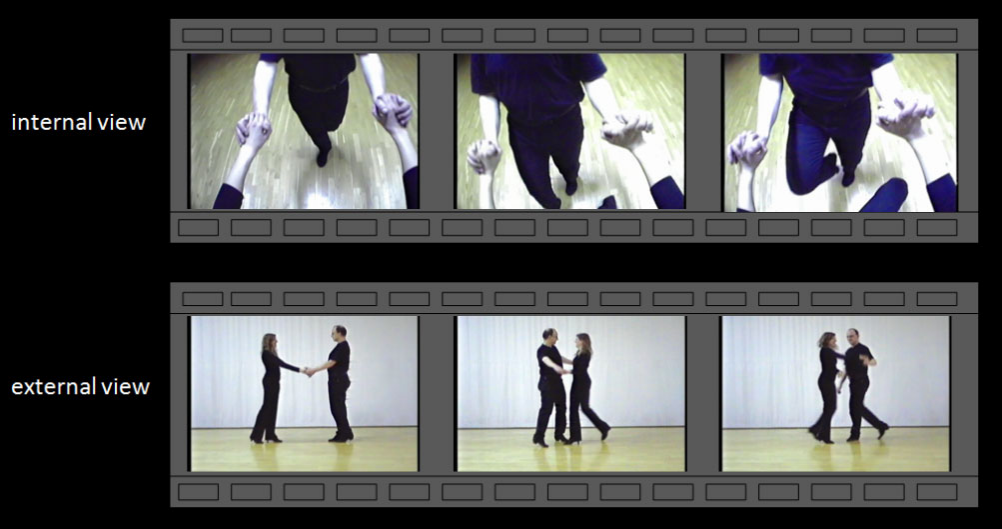
**Example of stimulation**. Example of stimulation (upper line: internal view, lower line: external view).

Four different types of stimuli were presented in a randomized order by a PC running Presentation software (Neurobehavioral Systems, Albany, USA) while participants underwent functional magnetic resonance scanning. The stimuli were projected onto a screen behind the scanner and viewed through a mirror attached to the head coil (visual field 188 mm in the horizontal and 168 mm in the vertical plane, rectangular aperture). All stimuli were videos of a ballroom dance scene: (1) videos from an internal viewpoint (IV), (2) videos from an external viewpoint (EV), (3) scrambled videos from an internal viewpoint (SIV), (4) scrambled videos from an external viewpoint (SEV). The videos showed a selection of Standard and Latin American ballroom dance (cha-cha, jive, rumba, samba). A total of 10 video sequences for each perspective were used. The dance videos were not added with music (Figure 2).

To get scrambled videos the videos from internal and external viewpoint were cut into rows and columns. The resulting cubes were then reordered in a randomized manner. The scrambled videos were subtracted from the IV and EV videos to exclude interfering activation (e.g., non-biological movement). 

In order to create the internal viewpoint stimuli, an experienced (having dancing experience for 10 years) female dancer wore a helmet camera (Montacor TVCCD-34COL, 752 × 582 pixels, 140 degree lens) on her forehead. External viewpoint stimuli were recorded with the same camera, at the same height, and at a distance of five meters from the dance scene. Identical dance sequences were danced during both recordings.

Before the experiment started, participants completed a questionnaire assessing their dance experience. Novices had little experience in ballroom dancing: They had attended no more than four dance classes in their life, and the last class was at least 4 years in the past. Experts were all skilled dancers with at least 5 years of continuous dance experience.

During the experiment, each trial started with a blank screen for 12 s. This was followed by an 18- to 25-s video. Durations of videos were identical in both the internal and the external viewing conditions. Participants were instructed to watch the stimuli carefully, because they would have to perform a test after the scanner session. Each stimulus class was presented 10 times and all participants observed all stimuli. The total scanner experiment lasted 32 min.

After the scanning session, participants completed a recognition test. This consisted of six test videos from an internal and six videos from an external viewpoint. Three videos from each category (IV and EV) had been shown during the scanner session. The participants had to judge whether or not they had observed the specific video.

### Image Acquisition and Analysis

Brain images were acquired using a 1.5 Tesla system (Siemens, Erlangen, Germany) with a standard head coil. A total of 805 T2*-weighted images were acquired (echoplanar imaging: repetition time = 2500 ms, echo time = 55 ms, matrix: 64 × 64). Structural image acquisition consisted of 160 T1-weighted sagittal images (1 mm slice thickness). All preprocessing analyses were carried out with SPM5 (Wellcome Department of Cognitive Neurology, London, UK). Volumes were realigned and unwarped, slice-time corrected, normalized to Montreal Neurological Institute (MNI) space, and smoothed with a 9-mm Gaussian isotropic kernel. All observation periods were modeled using a canonical hemodynamic response function with a duration matched to the video and picture presentation length. Data were high-pass filtered (cutoff = 256 s). Serial correlations were accounted for by an autoregressive (1) process. The four experimental conditions (IV, EV, SIV, SEV) were entered into the model. In the second-level analysis, a flexible factorial design was applied in SPM8 (Wellcome Department of Cognitive Neurology, London, UK).

In a first step, we contrasted each observation condition with the corresponding scrambled condition. The resulting *t *contrasts were fed into a conjunction analysis to detect which brain regions were activated in the mere observation of dance movements. The statistical threshold was set at *p *= 0.05, corrected for multiple comparisons using the familywise (FWE) error criterion. The *t *values of significant activations of the highest activated voxels were calculated and assigned to anatomical regions. All regions were mapped with maps based on cytoarchitectonic data [39].

Both t tests *(IV-SIV) - (EV-SEV*) and *(IV-SIV) + (EV-SEV) *were computed to compare novices and experts. The ventral and dorsal premotor cortex were chosen as regions of interest (ROIs) for these contrasts. Masks for small-volume correction were created using Eickhoff et al.'s [39] ANATOMY Toolbox implemented in SPM. As suggested by Rizzolatti et al. [5], the premotor cortex (BA6) was divided into ventral and dorsal premotor cortex at *Z *= 50. Only voxels with at least a 25% probability of belonging to the premotor cortex (BA6) were included in the masks. Significance was tested on a voxel level (α = 0.05, familywise error-corrected).

## Authors' contributions

SP carried out the fMRI study, performed the data analysis, and drafted the manuscript. BL participated in the design of the study and helped to draft the manuscript. RS participated in the design of the study and the data analysis. JM helped in the coordination and the draft of the manuscript. DV participated in the design and in the draft of the manuscript. KZ conceived the study as well as its design and helped to draft the manuscript. All authors read and approved the final manuscript.
